# Tuberculosis and Sarcoidosis Overlap: A Clinical Challenge From Diagnosis to Treatment

**DOI:** 10.7759/cureus.11662

**Published:** 2020-11-23

**Authors:** Ana Pedroso, Inês Ferreira, Teurai Chikura

**Affiliations:** 1 Internal Medicine Department, Hospital São Francisco Xavier, Lisboa, PRT; 2 Respiratory Medicine Department, Canterbury District Health Board, Christchurch, NZL

**Keywords:** tuberculosis, sarcoidosis, granulomata, immunosuppression, mycobacterium

## Abstract

Tuberculosis (TB) and sarcoidosis have clinical, immunologic, and radiologic similarities and the differential diagnosis is often a challenge. Some cases are described in which patients have both diseases concomitantly. There is a hypothesis that posits TB and sarcoidosis as being along the spectrum of the same disease. This has important implications for treatment decisions, since immunosuppression, which is a treatment for sarcoidosis, is undesirable in TB patients.

We are going to describe a clinical case of a TB patient who developed more severe symptoms during the course of TB treatment and, after excluding TB progression or resistance, he was diagnosed as probable sarcoidosis. He was started on immunosuppression, with great improvement, finishing the TB treatment completely asymptomatic.

## Introduction

Tuberculosis (TB) and sarcoidosis are chronic granulomatous diseases that share similar symptoms, immunologic processes and radiologic features.

TB is an infection caused by *Mycobacterium tuberculosis* (MTB) which affects around a quarter of the world population, but only a relatively small proportion (5-10%) of the people infected with MTB will develop TB disease during their lifetime. However, it is one of the top 10 causes of death worldwide and the leading cause of death from a single infectious agent [1].

Sarcoidosis is a multisystem granulomatous inflammatory disease with formation of non-caseating granulomas of unknown aetiology. Its incidence varies around the world, and is highest in Afro-Americans and Northern Europeans [2]. In New Zealand, the prevalence of sarcoidosis has been reported as 23-28/100,000 [3]. The symptoms also differ, for example, it appears that Maori and Pacific Island people have less pulmonary manifestations of sarcoidosis but more extra-pulmonary ones compared with European ancestry New Zealanders [4].

These two diseases were initially postulated to be part of the same disease, as they have similar clinical manifestations and histopathology. Tuberculosis can be a complication of treatment in sarcoidosis, but the two conditions rarely exist simultaneously. Nevertheless, some reports have been published about cases with simultaneous tuberculosis and sarcoidosis, from pulmonary [5-8] to extra-pulmonary presentations [6,9,10] and diverse sequences of presentations. Furthermore, several studies have shown the presence of MTB deoxyribonucleic acid (DNA) in a high proportion of tissue and bronchoalveolar lavage samples from patients with sarcoidosis. However, negative results have also been reported [11].

It has been suggested that mycobacterial antigens in genetically different predisposed hosts may be a cause of sarcoidosis [12,13]. Some authors then proposed that TB and sarcoidosis should be understood as different presentations along the spectrum of the same disease [14-16], but this is not yet firmly established.

This question has major implications for diagnosis and treatment. Reactivation of tuberculosis after corticosteroid treatment for sarcoidosis is an important concern, given the high prevalence of latent tuberculosis infection worldwide [1].

## Case presentation

A 35-year-old Indian man with no previous conditions or medication presented with a four-month history of increasing lethargy, general weakness, cough with creamy phlegm and specks of blood, followed by bilateral pleuritic chest pain. No fever was noted. He had been living in New Zealand for the last four years prior to presentation and had a clear chest radiogram four years before. There was no previous history of TB or infectious contacts. He travelled to India a year prior to his illness and stayed for a month. He had never smoked and has no alcohol habits.

**Figure 1:**
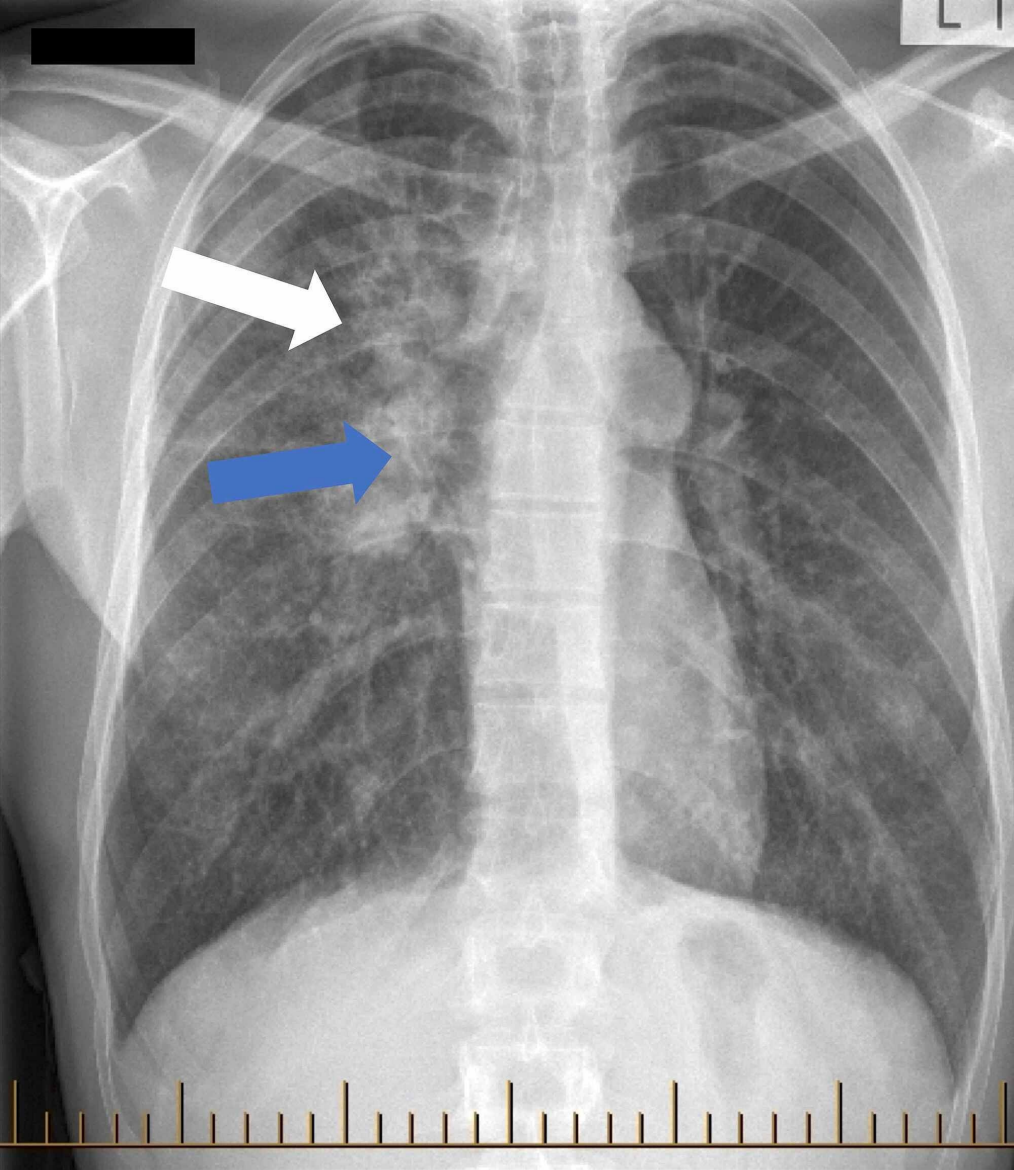
Chest radiogram showed extensive consolidation in the right upper lobe (white arrow) and right hilar lymphadenopathy (blue arrow).

A chest radiogram done for immigration purposes just prior to presentation showed extensive consolidation in the right upper lobe suspicious for TB (Figure 1) and he was immediately placed in community TB isolation. 

**Figure 2:**
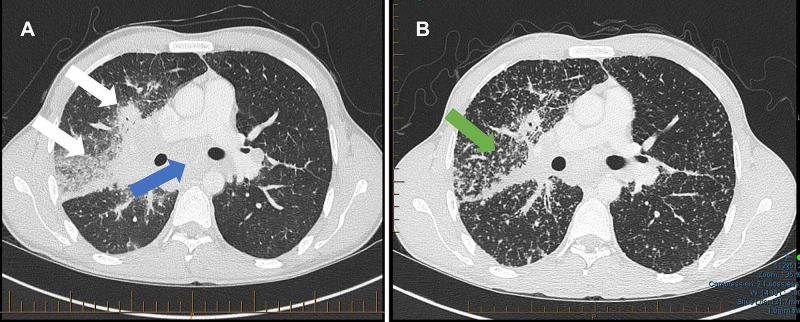
Chest CT scan A) at the diagnosis, showed right upper and middle zone parenchymal infiltrate (white arrows) and extensive mediastinal adenopathy (blue arrow); B) Worsening symptoms, showed a widespread miliary pattern (green arrow) with extensive involvement. CT - computed tomography

Further investigations were done: computed tomography (CT) showed right upper and middle zone parenchymal infiltrates and extensive mediastinal adenopathy (Figure 2A). Serial spontaneous sputum, bronchial washings and transbronchial lung biopsy were all MTB polymerase chain reaction (PCR) and mycobacterial culture negative. No other pathogens were identified. Transbronchial lung biopsy showed non-caseating granulomata, CD4/CD8 relation was not increased.

The diagnosis of TB was finally made from endobronchial ultrasound-guided fine-needle aspiration (EBUS-FNA) of sub-carinal lymph nodes, with a positive MTB PCR (Gene Xpert). The organism was fully drug-sensitive. Human Immunodeficiency Virus (HIV) and hepatitis C virus (HCV) infection was also excluded.

He was started on quadruple treatment with rifampicin/isoniazid 600/300 mg, pyrazinamide 1500mg, ethambutol 800 mg, pyridoxine 25 mg (once daily doses under directly observed therapy). Cholecalciferol supplement was also started because of vitamine D insufficiency.

A short time later he was admitted to hospital with hypercalcemia (corrected calcium of 3.6 mmol/L), felt likely to be secondary to tuberculosis and vitamin D treatment. Symptoms included significant fatigue. He was treated with intravenous fluid therapy and bisphosphonates and vitamin D was withheld. He also had cytolytic hepatitis presumed due to pyrazinamide, considering the liver tests returned to normal after suspension. He was started moxifloxacin 400 mg daily instead.

However, on the fifth month of treatment he kept feeling tired and a follow-up CT scan showed progressive abnormality compared to his initial CT. It showed a widespread miliary pattern with extensive involvement, clearly worse than the initial, although the right upper lobe consolidation had improved somewhat (Figure 2B). 

Considering his MTB was fully drug-sensitive and he had been on appropriate treatment, with adequate isoniazid levels confirmed, the differential for the CT changes would less likely be progressive TB. A second pathology, including paradoxical reaction to TB treatment and sarcoidosis, were considered. We repeated EBUS-FNA which confirmed negative MTB PCR, acid-fast bacilli (AFB) smear and mycobacterial culture. EBUS cytology showed mild granulomatous inflammation and no malignancy. Spirometry was normal.

The pulmonary appearances, the hypercalcaemia, markedly elevated serum angiotensin-converting enzyme (ACE) (206 IU/L) and florid radiological abnormality, as well as fatigue, suggested active pulmonary sarcoidosis. He had non-caseating granulomata on his first transbronchial lung biopsy, which also supports more the diagnosis of pulmonary sarcoidosis than tuberculosis. At this point, seven months into his treatment, it was decided to start systemic steroids (prednisone 0.5 mg/kg once daily weaning until two months after the end of TB treatment), with a rapid significant clinical and radiological improvement.

He finished the nine-month TB regimen as planned with complete recovery. A short time after completion of TB treatment it was possible to wean the prednisone treatment as well. Two months later, he stayed clinically well and chest radiogram remained clear.

## Discussion

TB and sarcoidosis are diseases with similar presentation and considered as having some common aetiology. As well as TB, HCV is also a causative factor. Infections have to be excluded before assuming the diagnosis of sarcoidosis.

However, even when TB is diagnosed, sarcoidosis must be considered if symptoms do not improve or get worse during the treatment of TB and there are no other factors that can explain the deterioration, such as antibiotic resistance, therapy nonadherence, paradoxical reaction to TB treatment or progressive disease.

The different hypothesis supporting that mycobacterial antigens in genetically predisposed hosts may cause sarcoidosis [12,13] and that TB and sarcoidosis should be understood as different presentations along the spectrum of the same disease [14-16] are controversial. However, they should be considered to better understand and approach cases such the one we present.

## Conclusions

This case shows how important it is to keep the differential diagnosis open in TB/sarcoidosis overlap and start immunosuppressive treatment when infective causes are excluded.

It can support the hypothesis of TB and sarcoidosis as part of the broad spectrum of the same disease and that mycobacterial antigens in genetic predisposed hosts may be a cause of sarcoidosis.

## References

[1] (2020). World Health Organization Global tuberculosis report 2019. https://apps.who.int/iris/handle/10665/329368.

[2] Newman LS, Rose CS, Maier LA (1997). Sarcoidosis. N Engl J Med.

[3] Mackay JB, Laing MC, Reid JD (1964). Sarcoidosis: some observations on present status, prevalence and treatment. N Z Med J.

[4] Wilsher ML, Young LM, Hopkins R, Corner M (2017). Characteristics of sarcoidosis in Maori and Pacific Islanders. Respirology.

[5] Carbonelli C, Giuffreda E, Palmiotti A (2017). Coexistent sarcoidosis and tuberculosis: a case report. Respiration.

[6] Mise K, Goic-Barisic I, Puizina-Ivic N, Barisic I, Tonkic M, Peric I (2010). A rare case of pulmonary tuberculosis with simultaneous pulmonary and skin sarcoidosis: a case report. Cases J.

[7] Papaetis GS, Pefanis A, Solomon S, Tsangarakis I, Orphanidou D, Achimastos A (2008). Asymptomatic stage I sarcoidosis complicated by pulmonary tuberculosis: a case report. J Med Case Rep.

[8] Piotrowski WJ, Górski P, Duda-Szymańska J, Kwiatkowska S (2014). Mycobacterium tuberculosis as a sarcoid factor? A case report of family sarcoidosis. Am J Case Rep.

[9] Luk A, Lee A, Ahn E, Soor GS, Ross HJ, Butany J (2010). Cardiac sarcoidosis: recurrent disease in a heart transplant patient following pulmonary tuberculosis infection. Can J Cardiol.

[10] Sarkar S, Saha K, Das CS (2010). Isolated tuberculous liver abscess in a patient with asymptomatic stage I sarcoidosis. Respir Care.

[11] Gupta D, Agarwal R, Aggarwal AN, Jindal SK (2007). Molecular evidence for the role of mycobacteria in sarcoidosis: a meta-analysis. Eur Respir J.

[12] Chen ES, Wahlström J, Song Z (2008). T cell responses to mycobacterial catalase-peroxidase profile a pathogenic antigen in systemic sarcoidosis. J Immunol.

[13] Dubaniewicz A, Dubaniewicz-Wybieralska M, Sternau A (2006). Mycobacterium tuberculosis complex and mycobacterial heat shock proteins in lymph node tissue from patients with pulmonary sarcoidosis. J Clin Microbiol.

[14] Agrawal R, Kee AR, Ang L (2016). Tuberculosis or sarcoidosis: opposite ends of the same disease spectrum?. Tuberculosis.

[15] Dubaniewicz A, Zimmermann A, Dudziak M, Typiak M, Skotarczak M (2013). Tuberculosis in the course of sarcoidosis treatment: is genotyping necessary for personalized therapy?. Expert Rev Clin Immunol.

[16] Gupta D, Agarwal R, Aggarwal AN, Jindal SK (2012). Sarcoidosis and tuberculosis: the same disease with different manifestations or similar manifestations of different disorders. Curr Opin Pulm Med.

